# Preservation vs. dissection of inferior pulmonary ligament for thoracoscopic upper lobectomy: a prospective randomized controlled trial

**DOI:** 10.1186/s12957-023-03190-8

**Published:** 2023-10-07

**Authors:** Jiekun Qian, Shixian Cai, Pinghua Lin, Wanzhong Chi, Chun Chen, Guobin Xu, Chi Xu, Weidong Wu, Wei Zheng, Bin Zheng

**Affiliations:** 1 Department of Thoracic Surgery, Fujian Medical University Union Hospital, Fuzhou, 350001 China; 2 Fujian Key Laboratory of Cardiothoracic Surgery (Fujian Medical University), Fuzhou, China; 3 Department of Thoracic Surgery, Jinjiang Hospital of Traditional Chinese Medicine, Quanzhou, China; 4 Department of Thoracic Surgery, Fuqing City Hospital, Fuzhou, China; 5 Department of Thoracic Surgery, Sanming Second Hospital, Sanming, China

**Keywords:** Thoracoscopic upper lobectomy, Inferior pulmonary ligament, Residual bronchial angle change

## Abstract

**Objectives:**

The proper procedure for inferior pulmonary ligament (IPL) during upper lobectomy remains a topic of debate. To address this matter, we carried out a trial comparing the clinical outcomes of IPL preservation versus IPL dissection during thoracoscopic upper lobectomy (TUL).

**Methods:**

Patients undergoing thoracoscopic left/right upper lobectomy (TLUL/TRUL) were assigned to either the dissection group (Group D) or the preservation group (Group P). Our primary objective was to quantify and compare the alterations in postoperative residual bronchial angle and lung volume changes between the two groups. Our secondary objective encompassed the assessment of various other intraoperative and postoperative outcomes.

**Results:**

Following adherence to the inclusion and exclusion criteria, we enrolled 100 patients (41 left and 59 right) in Group P and 108 patients (41 left and 67 right) in Group D for the study. Our findings revealed that in TLUL, Group P was able to reduce the degree of postoperative residual bronchial angle change (*P* < 0.05). Conversely, the situation was distinct for TRUL. We found no notable disparity between the two groups (*P* > 0.05) with regard to alterations in lung volume or the occurrence of postoperative complications—except for the duration of postoperative hospital stay (*P* < 0.05).

**Conclusions:**

Our study suggests IPL preservation especially for TLUL when compared to TRUL, which have important implications for the clinical management of patients undergoing upper lobectomy.

## Introduction

According to the latest cancer statistics, lung cancer currently holds the top position in China for both incidence and mortality among malignant tumors [1]. Projections suggest that the number of lung cancer deaths worldwide will continue to rise significantly over the next 10–20 years [2]. Hence, it is imperative to introduce effective interventions without delay. Fortunately, with the widespread adoption of low-dose lung CT scans and the enhancement of public health awareness, the proportion of early-stage lung cancer detections has steadily risen compared to previous years. In such instances, timely surgical intervention becomes pivotal for achieving a favorable prognosis. According to current international guidelines, radical resection is the recommended local treatment for early-stage non-small cell lung cancer (NSCLC), with thoracoscopic anatomical lobectomy being the standard operation [3].

There is evidence indicating that a majority of lesions are located in the upper lobes across all types of lung cancer [4]. Most thoracoscopic upper lobectomy (TUL) procedures are performed due to the high incidence of lung cancer detected in the upper lobe. Previous studies have shown that many thoracic surgeons tend to divide the inferior pulmonary ligament (IPL) during upper lobectomy [5, 6]. In theory, a residual cavity is bound to form after the removal of the upper lobe during the operation, and this represents a risk factor for postoperative complications such as pleural effusion and pulmonary infection. Traditionally, dissecting IPL during surgery is believed to mitigate the postoperative restriction of the lower lobe’s range of motion, thus enhancing its expansion and filling the residual cavity, which can reduce postoperative atelectasis and pleural effusion [7, 8]. However, IPL may have essential functions beyond lung lobe immobilization, including roles in pleural fluid secretion and reabsorption [8]. There is currently no sufficient evidence to prove that isolating IPL during surgery can improve patient prognosis, and there is still great controversy about whether this step should be performed. Different doctors may opt for different surgical approaches, often relying on their individual clinical experience, as there is no standardized consensus [9]. Previous studies have identified that patients undergoing upper lobectomy may experience varying degrees of residual bronchial tortuosity and stenosis following the surgical procedure [10]. Further analysis has indicated that these problems could be attributed to the excessive redistribution of residual lobes resulting from the dissection of the inferior pulmonary ligament (IPL) during surgery. Furthermore, the residual bronchial malformation may lead to postoperative complications such as atelectasis, lung infections, reduced lung function, persistent refractory dry cough, shortness of breath, and other adverse effects.

To explore the best surgical plan, we designed a multicenter prospective randomized controlled study based on mature experience in TUL. We investigated and compared the distinct clinical outcomes of IPL dissection and IPL preservation in patients with TUL for various indications, not limited to lung cancer. Our aim was to offer specific evidence to guide clinical decision-making regarding operative techniques.

## Patients and methods

### Research object and purpose

This study is a multicenter, prospective, open-label, controlled research that enrolled patients admitted to Fujian Medical University Union Hospital, Fuqing City Hospital, and Sanming Second Hospital between March 2020 and March 2022 and who were scheduled to undergo TUL. The patients were randomly assigned to either the dissection group (Group D) or the preservation group (Group P) using the random number method, based on predetermined criteria.

### Patient cohort

#### Inclusion criteria

1. Eighteen years old < age < 70 years old
2. Meet the indications for TUL
3. Preoperative pulmonary function test: FEV1 > 1 L and FEV1 > 60% of the predicted value
4. Preoperative ECOG score of 0–1, preoperative ASA scores 1–2

#### Exclusion or rejection criteria

1. Inferior mediastinal lymphadenopathy was found in preoperative screening
2. Found that a larger-scale operation is required due to multiple lesions
3. History of thoracic surgery
4. Severe thoracic adhesions found during surgery
5. Lost to follow-up

### Randomization

Upon obtaining informed consent, patients were enrolled in an electronic data capture system by a research staff member and were given a unique study number prior to randomization. Patients were then randomized in a 1:1 ratio to either Group P or Group D. Following randomization, the allocated operative procedure was communicated to both the patient and surgeon. Patients and all investigators were unmasked to the treatment assignment.

### Surgical technique

All enrolled patients underwent TUL with both groups receiving the same surgery procedure, except for the treatment of IPL on the surgical side and the lymph nodes around it. With the exception of two patients (one with a benign nodule and another with sclerosing pneumocytoma), all enrolled individuals underwent systematic lymph node dissection as much as possible, including pulmonary ligament lymph nodes (9th LNs). In Group P, the 9th LNs were removed only at the root of the inferior pulmonary vein. In Group D, IPL was separated with an electrocoagulation hook or ultrasonic knife. Paraesophageal lymph nodes and the 9th LNs were further dissected until the inferior pulmonary vein was exposed. It should be noted that at the end of the operation, two chest tubes will be placed. One is designated as closed thoracic drainage tube, characterized by its relatively robust diameter and elevated placement. This tube primarily serves to facilitate the drainage of air. If no significant postoperative air leakage is observed, this tube is eventually removed. The other tube, labeled as disposable drainage catheter, features a comparatively slender diameter and is positioned at a lower level. Its primary function is to drain postoperative effusion. When the quantity of postoperative effusion remains minimal (less than 150 ml/day), this tube is subsequently removed. All operations were performed by experienced thoracic surgeons in our medical centers.

### Follow-up

Enrolled patients were followed up until at least 6 months postoperatively.

### Primary study endpoint

#### Changes in bronchial angle

**Fig. 1:**
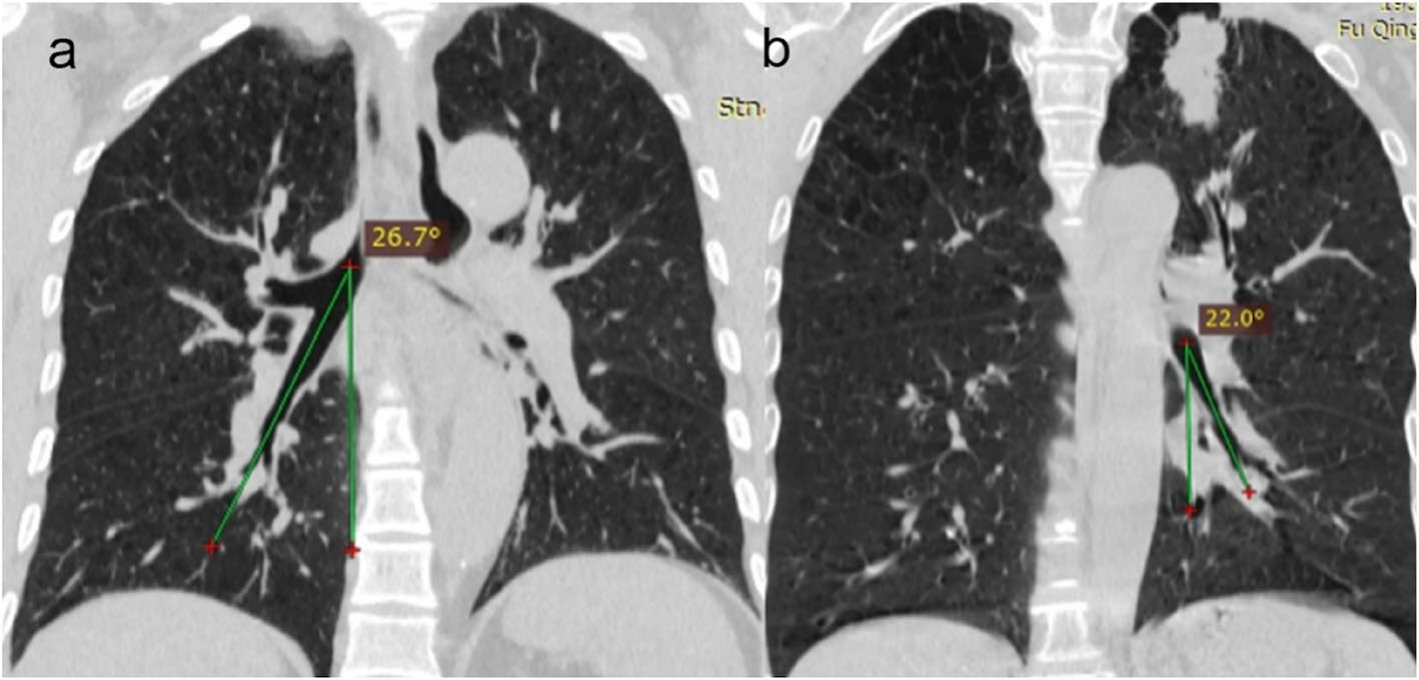
Measurement of bronchial angles. **a** The right bronchial angle: formed by the right intermediate bronchus and the midline of the trachea (extended line). **b** The left bronchial angle: formed by the left lower lobe bronchus and the midline of the trachea (extended line)

The angle created by the left lower lobe bronchus and the midline of the trachea (extended line), as well as the angle formed by the right intermediate bronchus and the midline of the trachea (extended line), were evaluated using chest CT coronal images (Fig. 1). These measurements were conducted by an experienced thoracic surgeon and a proficient radiologist both prior to the surgical procedure and at the 3- and 6-month postoperative intervals.

#### Changes in lung volume

**Fig. 2:**
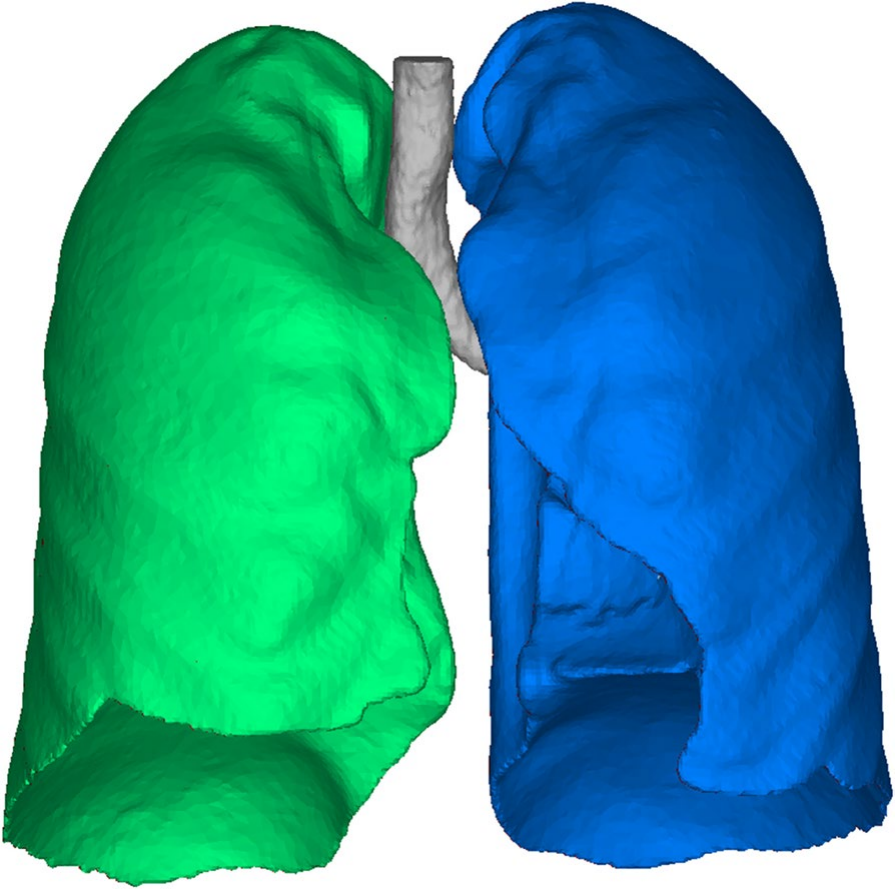
Three-dimensional lung volumetry. Semiautomated measurements of three-dimensional lung volumes were carried out using a reconstruction software (Mimics Research 21.0), utilizing Hounsfield unit (HU) values within the range of − 1024 to 500 HU

Pulmonary volumetry is a noninvasive, easy, and fast measurement technique that uses 3D reconstruction software to analyze patient CT scans and calculate each patient’s lung volume. We performed semiautomated measurements of three-dimensional lung volumes using a reconstruction software (Mimics Research 21.0), with the range of Hounsfield Unit (HU) values set from − 1024 to − 500 HU (Fig. 2). These measurements were performed both prior to and at the 3- and 6-month intervals following the surgical procedure, and they were meticulously documented by members of our team. The discrepancies between postoperative and preoperative volumes were calculated for further analysis.

### Secondary study endpoints

#### Postoperative complications

Incidence of the postoperative residual cavity, pleural effusion, lung infection, and atrial fibrillation was recorded during the follow-up procedure.

#### Intraoperative and postoperative clinical data

Pathological cancer stage, histological diagnosis, operation time, postoperative hospital stay, closed thoracic drainage tube retention time, and disposable drainage catheter retention time were recorded during the follow-up procedure.

### Statistical methods

SPSS25.0 was used to process the collected data, continuous variables are expressed as mean (SD), and categorical variables are expressed as frequency. The Shapiro–Wilk test was used to confirm the normality of the variables. A Student’s *t*-test was used to compare the continuous variables that conformed to the normal distribution between the two groups; the categorical variables were tested by the chi-squared test or Fisher’s exact test. The test level is valid at *P* < 0.05.

## Results

### Patient demographics and perioperative clinical characteristics

**Fig. 3:**
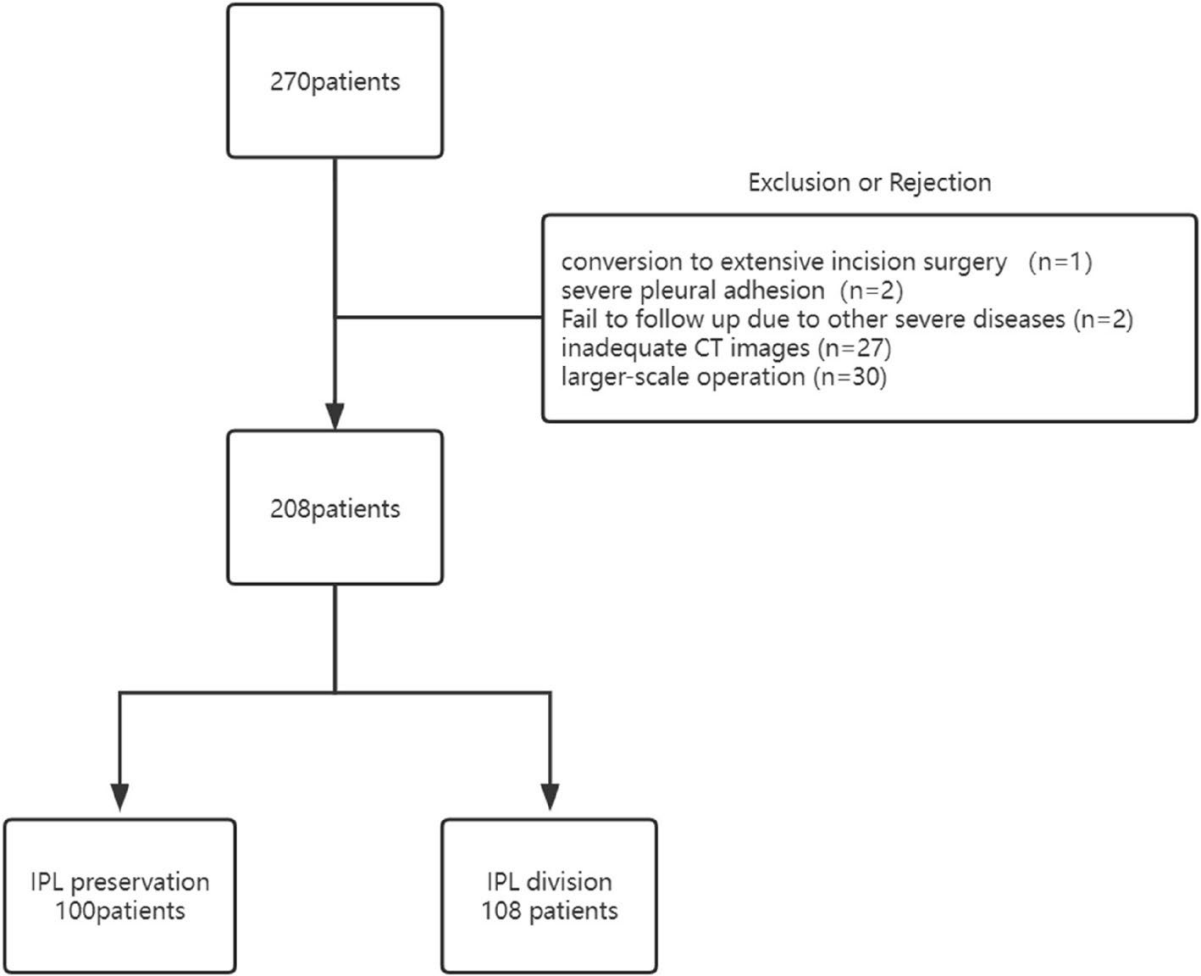
Flowchart of patient selection. Initially, 270 patients were enrolled in this study before surgery. After exclusions, 62 patients were removed from the study, resulting in a final inclusion of 208 patients

According to the eligibility criteria, 270 patients were initially enrolled in this study before surgery (Fig. 3). All patients underwent thoracoscopic left/right upper lobe resection as expected. However, during the process, 62 patients were excluded. Ultimately, 208 patients were included in the study, with 100 patients in Group P (41 left and 59 right) and 108 patients in Group D (41 left and 67 right). There were no significant differences in sex, age, preoperative comorbidities, pathological cancer stages, and pathological types between the two groups (*P* > 0.05) (Tables 1 and 2).

**Table 1:** Baseline characteristics between two groups (pre-op)

Variables	Group P (*n* = 100)	Group D (*n* = 108)	*P*
Operation side			0.654
Left	41	41	
Right	59	67	
Sex			0.455
Male	44	42	
Female	56	66	
Age, years (x ± s)	59.08 ± 9.82	58.45 ± 10.74	0.662
Preoperative comorbidities			
Hypertension	24	27	1.000
Diabetes	6	10	0.378
Coronary heart disease	6	4	0.653

**Table 2:** Baseline characteristics between two groups (post-op)

Variables	Group P (*n* = 100)	Group D (*n* = 108)	*P*
Pathological cancer stage			0.582
IA1	20	13	
IA2	34	43	
IA3	23	24	
IB	5	9	
IIA	1	1	
IIB	4	4	
IIIA	10	7	
Unstaged	3	7	
Histological diagnosis			0.801
Squamous cell carcinoma	2	2	
Adenocarcinoma	90	95	
Mucinous adenocarcinoma	3	4	
Adenosquamous carcinoma	1	1	
Benign nodule	1	1	
Sclerosing pneumocytoma	0	1	
Lymphoepithelioma-like carcinoma	1	0	
Lymphoma	0	2	
Mucoepidermoid carcinoma	0	1	
Myoepithelial carcinoma	0	1	
Pleomorphic carcinoma	2	0	

### Residual bronchial angel changes

At 3 months after thoracoscopic left upper lobectomy (TLUL), the changes in the residual bronchial angles of Group P and Group D were 50.73° ± 20.53° and 61.24° ± 17.57°, respectively, and there was a statistically significant difference between the groups (*P* < 0.05). At 3 months after thoracoscopic right upper lobectomy (TRUL), the angle change of the residual bronchus in Group P was 37.45° ± 17.11°, lower than 44.16° ± 21.67° of Group D, but without a significant difference (*P* > 0.05) (Table 3).

**Table 3:** Change in bronchial angles between two groups (°, x ± s)

Variable	Group P	Group D	*P*
Overall			
Postoperative time, months			
3	42.89 ± 19.62	50.64 ± 21.78	0.008
6	42.83 ± 17.84	51.08 ± 20.10	0.002
Left lung			
Postoperative time, months			
3	50.73 ± 20.53	61.24 ± 17.57	0.015
6	48.99 ± 17.30	63.01 ± 13.60	< 0.001
Right lung			
Postoperative time, months			
3	37.45 ± 17.11	44.16 ± 21.67	0.058
6	38.55 ± 17.08	43.78 ± 20.01	0.120

At 6 months after TLUL, the changes in the residual bronchial angles of Group P and Group D were 48.99° ± 17.30° and 63.01° ± 13.60°, respectively, and there was a statistically significant difference between the groups (*P* < 0.001). At 6 months after TRUL, the angle change of the residual bronchus in Group P was 38.55° ± 17.08°, lower than 43.78° ± 20.01° of Group D, but without a significant difference (*P* > 0.05) (Table 3).

### Lung volume changes

Three and 6 months after TLUL, the changes in lung volume on the operative side were not significantly different between Group P and Group D (− 504.39 ± 465.54 ml vs. − 418.06 ± 521.90 ml at 3 months; − 487.02 ± 324.63 ml vs. − 422.56 ± 317.80 ml at 6 months, both *P* > 0.05). Similarly, for patients undergoing TRUL, there was no significant difference in lung volume changes on the operative side between Group P and Group D (− 370.50 ± 405.91 ml vs. − 367.96 ± 426.82 ml at 3 months; − 439.01 ± 366.68 ml vs. − 470.02 ± 372.46 ml at 6 months, both *P* > 0.05). Moreover, there was no significant difference in the changes of lung volume on the healthy side between the two groups at either time point (*P* > 0.05) (Table 4).

**Table 4:** Change in lung volume between two groups (ml, x ± s)

Variable	Group P	Group D	P
Left lung			
Measuring position			
Left			
Postoperative time, months			
3	− 504.39 ± 465.54	− 418.06 ± 521.90	0.441
6	− 487.02 ± 324.63	− 422.56 ± 317.80	0.373
Right			
Postoperative time, months			
3	62.42 ± 359.97	− 8.50 ± 533.44	0.495
6	66.03 ± 300.63	170.05 ± 346.10	0.159
Right lung			
Measuring position			
Left			
Postoperative time, months			
3	70.95 ± 263.84	61.30 ± 422.10	0.878
6	56.71 ± 391.43	− 2.36 ± 391.71	0.395
Right			
Postoperative time, months			
3	− 370.50 ± 405.91	− 367.96 ± 426.82	0.973
6	− 439.01 ± 366.68	− 470.02 ± 372.46	0.636

### Complications and other clinical data

There were 22 patients (32.8%) with postoperative pleural effusion in Group D on the right side, which was higher than 12 (20.3%) in Group P, but without a significant difference (*P* = 0.115). While on the left side, there were 16 patients (39.0%) with postoperative pleural effusion in Group D, the same as 16 (39.0%) in Group P (*P* = 1.000). In Group P, four patients (4.0%) developed apical dead space after surgery, 22 (22.0%) developed pulmonary infection after surgery, and 5 (5.0%) developed atrial fibrillation after surgery. In Group D, there were 7 (6.5%) patients with postoperative apical dead space, 24 (22.2%) with postoperative pulmonary infection, and 4 (3.7%) with postoperative atrial fibrillation. Through comparison and analysis, it was found that there was no statistically significant difference in the incidence of the above postoperative complications between the two groups (*P*-values were 0.424, 0.969, 0.741, respectively) (Table 5).

**Table 5:** Comparison of complications and other clinical data

Variable	Group P	Group D	P
Overall			
Apical dead space	4 (4.0%)	7 (6.5%)	0.424
Pleural effusion	28 (28.0%)	38 (35.2%)	0.266
Pulmonary infection	22 (22.0%)	24 (22.2%)	0.969
Atrial fibrillation	5 (5.0%)	4 (3.7%)	0.741
Operation time, min (x ± s)	146.09 ± 42.21	146.73 ± 38.16	0.909
Postoperative hospital stay, d (x ± s)	4.80 ± 1.94	5.66 ± 3.90	0.044
Closed thoracic drainage tube retention time, d (x ± s)	2.12 ± 1.17	2.40 ± 1.80	0.185
Disposable drainage catheter retention time, d (x ± s)	4.55 ± 1.94	5.14 ± 3.06	0.097
Left lung			
Apical dead space	1 (2.4%)	2 (4.9%)	1.000
Pleural effusion	16 (39.0%)	16 (39.0%)	1.000
Pulmonary infection	7 (17.1%)	7 (17.1%)	1.000
Atrial fibrillation	3 (7.3%)	2 (4.9%)	1.000
Operation time, min (x ± s)	155.98 ± 38.99	142.23 ± 31.41	0.082
Postoperative hospital stay, d (x ± s)	5.17 ± 2.22	5.29 ± 2.86	0.830
Closed thoracic drainage tube retention time, d (x ± s)	2.24 ± 1.55	2.95 ± 2.65	0.145
Disposable drainage catheter retention time, d (x ± s)	4.61 ± 2.14	4.83 ± 2.67	0.683
Right lung			
Apical dead space	3 (5.1%)	5 (7.5%)	0.722
Pleural effusion	12 (20.3%)	22 (32.8%)	0.115
Pulmonary infection	15 (25.4%)	17 (25.4%)	0.995
Atrial fibrillation	2 (3.4%)	2 (3.0%)	1.000
Operation time, min (x ± s)	139.22 ± 43.32	149.48 ± 41.75	0.178
Postoperative hospital stay, d (x ± s)	4.54 ± 1.68	5.88 ± 4.42	0.024
Closed thoracic drainage tube retention time, d (x ± s)	2.03 ± 0.81	2.06 ± 0.83	0.861
Disposable drainage catheter retention time, d (x ± s)	4.51 ± 1.80	5.33 ± 3.28	0.091

Patients in Group P had a shorter postoperative stay (4.54 ± 1.68 days) on the right side compared to those in Group D (5.88 ± 4.42 days) (*P* = 0.024). The left side’s postoperative stay (d) was 5.17 ± 2.22 in Group P and 5.29 ± 2.86 in Group D (*p* = 0.830). The duration of operation (min) is 146.09 ± 42.21 in Group P and 146.73 ± 38.16 in Group D (*p* = 0.909). The Closed thoracic drainage tube retention time(d) is 2.12 ± 1.17 in Group P and 2.40 ± 1.80 in Group D (*p* = 0.185). The disposable drainage catheter retention time (d) is 4.55 ± 1.94 in Group P and 5.14 ± 3.06 in Group D (*p* = 0.097) (Table 5).

## Discussion

The division of IPL during TUL remains a subject of ongoing debate. Historically, numerous thoracic surgeons held the belief that dissecting IPL during upper lobectomy could promote the expansion of the residual lobe, potentially reducing the postoperative thoracic dead space volume and preventing complications like atelectasis, pleural effusion, and empyema [5, 7]. Nonetheless, certain researchers have voiced concerns that separating IPL may have a contrary effect, potentially resulting in severe postoperative complications, including bronchial distortion stemming from excessive redistribution of the residual lung [8, 10, 11]. A previous study conducted in Japan, using a questionnaire-based approach, revealed that 69% of doctors in Japanese hospitals preferred to preserve IPL during upper lobectomy. These doctors believed that maintaining the structural integrity of IPL could reduce the occurrence of postoperative bronchial distortion and stenosis. However, they also acknowledged that this approach might elevate the risk of postoperative pleural effusion and pleural infection [9].

Previous literature has shown that about 90% of patients can recover their lung function to the expected value within 3–6 months after surgery [12]. This recovery is attributed to compensatory adaptation, which necessitates appropriate bronchial remodeling. However, excessive bronchial remodeling can lead to severe bronchial structural deformities, adversely affecting postoperative compensatory adaptation and subsequently impacting the recovery of lung function. Therefore, preventing bronchial distortion after upper lobectomy becomes paramount in mitigating postoperative declines in pulmonary function and enhancing the long-term postoperative quality of life for patients. The question of whether dividing IPL during upper lobectomy might lead to excessive remodeling of the residual bronchus remains uncertain. Following IPL dissection, the lower lung loses the stabilizing structure it provides, making it susceptible to upward deviation and potentially causing postoperative bronchial distortion. Such maladaptive remodeling may disrupt postoperative compensatory adaptation and further hinder the recovery of lung function. Historically, numerous studies have delved into the optimal IPL management during upper lobectomy, focusing on bronchial structural considerations. Seok Y. et al. [13] found that the left lower lung bronchus would sometimes twist severely upwards or even into a U shape after left upper lung resection. Van Leuven M. et al. [14] reported two cases of severe residual bronchial distortion after upper lobectomy, with one patient experiencing residual atelectasis. Both patients were relieved by bronchial stent placement. Ueda et al. [10] employed 3D imaging technology to examine postoperative bronchial distortion in 50 patients who underwent upper lobectomy, and the results revealed that nearly half of them experienced postoperative bronchial distortion. They also identified a correlation between postoperative bronchial distortion and the persistence of cough and shortness of breath after surgery. In a randomized clinical trial on 35 patients with upper lung adenocarcinoma, Matsuoka et al. [6] found no significant difference in the shape of the residual bronchus between IPL preservation and IPL division on chest radiographs. Pu Liang et al. [15] included 72 patients with NSCLC who underwent upper lobectomy and observed a significantly greater change in the main bronchial angle of the left lung compared to the right lung’s main bronchus following IPL isolation. Additionally, Sundaramoorei et al. [16] have documented a rare cause of dyspnea following upper lobectomy, which they have termed “post-lobectomy bronchomalacia.” During bronchoscopy, they observed the collapse of the right main bronchus lumen during expiration, returning to its normal state during inspiration. This phenomenon might arise from the re-expansion of the residual lung after upper lobectomy, exerting external pressure on the bronchus. Given that the bronchus contains cartilage, prolonged external pressure can potentially result in permanent deformity, leading to the loss of bronchial mechanical support and bronchial softening. In our study, we observed significant differences in the angle changes of the residual bronchus between Group D and Group P both 3 and 6 months after TLUL, indicating that the dissection of IPL during the procedure might lead to excessive displacement of the postoperative residual bronchus. While we also observed a higher change in residual bronchial angle in Group D compared to Group P after TRUL, this difference was not statistically significant. The left upper lobe’s anatomical equivalence to the right upper lobe and right middle lobe, along with its larger proportion of the chest cavity, provides greater potential for the re-expansion of the remaining lung following surgery. Additionally, the left side may exhibit more significant compensatory diaphragmatic mobility than the right side. Consequently, the left lower bronchus may experience a more pronounced upward pull, resulting in a larger angle formation.

Pulmonary volumetry enables objective and accurate observation of postoperative lung re-expansion. Moon et al. [17] demonstrated that Group D experienced greater postoperative lung volume loss after LUL compared to Group P. In contrast, our study did not find a significant difference in pre- and postoperative lung volume change between Group P and Group D for both the left and right sides. It is conceivable that mild residual bronchial distortion may not exert a substantial influence on the recovery of lung volume, as the recovery process is subject to numerous contributing factors.

The conventional view suggests that IPL preservation during upper lobectomy can lead to various postoperative complications such as apical dead space, pulmonary atelectasis, pleural effusion, and other issues [9]. Thoracic surgeons who advocate for the dissection of IPL during upper lobectomy often contend that preserving IPL can result in postoperative dead space, potentially leading to pleural effusion and, in some cases, more severe complications. However, Matsuoka et al. [6] reported that IPL division did not significantly reduce the postoperative dead space rate for either RUL or LUL. Studies by Kim et al. [18] and Seok Y. et al. [19] found no significant difference in the occurrence of delayed pleural effusion, postoperative chest tube retention time, and other postoperative indicators between the preservation and dissection groups, except for excessive residual bronchial deviation associated with IPL dissection. Our study found no significant difference between Group P and Group D in the incidence of complications such as apical dead space, pleural effusion, pulmonary infection, and atrial fibrillation, as well as in other clinical data such as operation time, the closed thoracic drainage tube time, and disposable drainage catheter retention time. These findings run counter to the theoretical assumption that IPL preservation can lead to complications and further emphasize the safety of preserving IPL.

Since systematic mediastinal lymph node dissection is recommended as the standard procedure following lobectomy for resectable lung cancer, IPL is often divided to enable a more thorough lymph node dissection that includes the lower mediastinal lymph nodes. With the wide application of low-dose spiral CT, an increasing number of individuals are being screened for early-stage lung cancer through routine physical examinations [20]. Studies have indicated that lower mediastinal lymph node dissection may not be necessary for early-stage lung cancer, as upper lobe tumors seldom metastasize to the lower mediastinal lymph nodes [20, 21]. Other pertinent studies [22–25] have demonstrated that selective lymph node dissection, guided by tumor location, provides equivalent survival benefits for early-stage lung cancer as compared to systematic lymph node dissection. Moreover, selective lymph node dissection can lead to shorter surgical durations and reduced surgical trauma. In this study, we only dissected the 9th LNs at the root of the inferior pulmonary vein in Group P to protect the integrity of IPL. Nevertheless, in cases where patients exhibit inferior mediastinal lymph node enlargement, it is still advisable to consider dividing IPL to facilitate a more comprehensive lymph node dissection. This approach aims to prevent any compromise to surgical effectiveness and postoperative pathological staging.

The strength of this trial lies in its status as the first multicenter prospective randomized controlled study investigating the management of IPL during TUL, thus providing a valuable reference for the procedure. However, there are still several limitations to this study. Firstly, the participating surgeons were drawn from three different medical centers, which might have resulted in slight variations in their surgical techniques. Nevertheless, these surgeons had extensive experience and had shared their surgical techniques for an extended period, ensuring a high degree of similarity in surgical approaches across institutions. Secondly, this study exclusively focuses on objective indicators and did not record subjective symptoms, such as postoperative pain, persistent cough, and shortness of breath, due to their difficulty in quantification. Thirdly, we did not extensively record the preoperative FEV1 and ASA/ECOG scores, potentially introducing reporting bias. Nonetheless, it is essential to emphasize that all enrolled patients met the inclusion criteria and fulfilled the necessary physical requirements for surgery. Lastly, to minimize bias, this study exclusively enrolled patients who underwent thoracoscopic surgery, which means the findings may not be directly applicable to open surgery cases.

## Conclusion

In the context of TUL, the resection of IPL is deemed unnecessary, particularly in cases of TLUL. IPL resection offers no discernible advantages and instead contributes to an increase in postoperative bronchial tortuosity in TLUL.

## Data Availability

The data supporting the findings of this study are available from the corresponding author upon reasonable request.

## Abbreviations

IPL: Inferior pulmonary ligament
TUL: Thoracoscopic upper lobectomy
TLUL: Thoracoscopic left upper lobectomy
TRUL: Thoracoscopic right upper lobectomy
Group D: The dissection group
Group P: The preservation group
NSCLC: Non-small cell lung cancer
9th LNs: Pulmonary ligament lymph nodes

## References

[1] Chen W, Sun K, Zheng R (2018). Cancer incidence and mortality in China, 2014. Chin J Cancer Res.

[2] Didkowska J, Wojciechowska U, Mańczuk M, et al. Lung cancer epidemiology: contemporary and future challenges worldwide. Ann Transl Med. 2016;4(8):150. 10.21037/atm.2016.03.11 PMC4860480 27195268

[3] Ettinger D S, Wood D E, Aisner D L, et al. Non–small cell lung cancer, version 3.2022, NCCN clinical practice guidelines in oncology. J Natl Compr Cancer Netw. 2022;20(5):497–530. 10.6004/jnccn.2022.0025 35545176

[4] Watanabe S, Asamura H, Suzuki K (2005). The new strategy of selective nodal dissection for lung cancer based on segment-specific patterns of nodal spread. Interact Cardiovasc Thorac Surg.

[5] Khanbhai M, Dunning J, Yap KH (2013). Dissection of the pulmonary ligament during upper lobectomy: is it necessary?. Interact Cardiovasc Thorac Surg.

[6] Matsuoka H, Nakamura H, Nishio W (2004). Division of the pulmonary ligament after upper lobectomy is less effective for the obliteration of dead space than leaving it intact. Surg Today.

[7] Kim CW, Godelman A, Jain VR (2011). Postlobectomy chest radiographic changes: a quantitative analysis. Can Assoc Radiol J.

[8] Riquet M, Barthes FLP, Souilamas R (2002). Thoracic duct tributaries from intrathoracic organs. Ann Thorac Surg.

[9] Usuda K, Sagawa M, Aikawa H (2010). Do Japanese thoracic surgeons think that dissection of the pulmonary ligament is necessary after an upper lobectomy?. Surg Today.

[10] Ueda K, Tanaka T, Hayashi M (2012). Clinical ramifications of bronchial kink after upper lobectomy. Ann Thorac Surg.

[11] Terzi A, Furlan G, Magnanelli G (1994). Chylothorax after pleuro-pulmonary surgery: a rare but unavoidable complication. Thorac Cardiovasc Surg.

[12] Bolliger CT, Jordan P, Soler M (1996). Pulmonary function and exercise capacity after lung resection. Eur Respir J.

[13] Seok Y, Cho S, Lee JY (2014). The effect of postoperative change in bronchial angle on postoperative pulmonary function after upper lobectomy in lung cancer patients. Interact Cardiovasc Thorac Surg.

[14] Van Leuven M, Clayman JA, Snow N (1999). Bronchial obstruction after upper lobectomy: kinked bronchus relieved by stenting. Ann Thorac Surg.

[15] Bu L, Yang AR, Peng H (2016). Dividing inferior pulmonary ligament may change the bronchial angle. J Surg Res.

[16] Sundaramoorthi T, Hashim S, Dillon P (2004). An unusual cause of breathlessness after lobectomy for lung cancer. Ann Thorac Surg.

[17] Moon DH, Park CH, Jung JH (2021). Inferior pulmonary ligament division may be unnecessary during left upper lobectomy: effects on lung volume, bronchial angle and bronchial tortuosity. J Clin Med.

[18] Kim DH, Moon DH, Kim HR (2019). Effect of inferior pulmonary ligament division on residual lung volume and function after a right upper lobectomy. Interact Cardiovasc Thorac Surg.

[19] Seok Y, Yi E, Cho S (2015). Perioperative outcomes of upper lobectomy according to preservation or division of the inferior pulmonary ligament. J Thorac Dis.

[20] Shimada Y, Saji H, Kakihana M (2012). Retrospective analysis of nodal spread patterns according to tumor location in pathological N2 non-small cell lung cancer. World J Surg.

[21] Cerfolio RJ, Bryant AS (2006). Distribution and likelihood of lymph node metastasis based on the lobar location of nonsmall-cell lung cancer. Ann Thorac Surg.

[22] Henschke CI, McCauley DI, Yankelevitz DF (2001). Early lung cancer action project: a summary of the findings on baseline screening. Oncologist.

[23] Su S, Scott WJ, Allen MS (2014). Patterns of survival and recurrence after surgical treatment of early stage non–small cell lung carcinoma in the ACOSOG Z0030 (ALLIANCE) trial. J Thorac Cardiovasc Surg.

[24] Han H, Chen H (2017). Selective lymph node dissection in early-stage non-small cell lung cancer. J Thorac Dis.

[25] Ishiguro F, Matsuo K, Fukui T (2010). Effect of selective lymph node dissection based on patterns of lobe-specific lymph node metastases on patient outcome in patients with resectable non–small cell lung cancer: a large-scale retrospective cohort study applying a propensity score. J Thorac Cardiovasc Surg.

